# Effects of a variable light intensity lighting program on the welfare and performance of commercial broiler chickens

**DOI:** 10.3389/fphys.2023.1059055

**Published:** 2023-02-24

**Authors:** Seong W. Kang, Karen D. Christensen, Michael T. Kidd Jr, Sara K. Orlowski, James Clark

**Affiliations:** ^1^ Department of Poultry Science, Center of Excellence for Poultry Science, University of AR, Fayetteville, AR, United States; ^2^ Tyson Foods, Inc, Springdale, AR, United States

**Keywords:** variable light intensity, broilers, welfare, serotonin, dopamine, BDNF, melanopsin

## Abstract

Our previous variable-light intensity lighting program studies indicate the light intensity preference behavior of broilers for their daily activity including eating and resting. To evaluate the effects of variable-light intensity lighting program on performance and welfare of broilers, four commercial trials were conducted for looking at behaviors, mortality, leg-health, performance, and brain welfare indicator genes including tryptophan hydroxylase 2 and tyrosine hydroxylase (TH), glucocorticoid receptor (GR), brain-derived neurotropic factor (BDNF), and melanopsin (Opn4) gene expression. One-day-old broilers were housed in four commercial broiler houses. Each quadrant (section) of the house was placed with 4,800 chicks. A total of four lighting programs began on day 7 with 5 lux (lx), 20 lx, natural light (NL, 480 lx), and variable light (2–5/40 lx) using LED lights on a 16L:8D photoperiod. In the variable-light house, the number of dustbathing holes was significantly higher than that in natural-light houses and 5-lx and 20-lx houses. Daily physical activities, footpad condition, fear response to novel objects, body weight, feed conversion ratio, and the number of leg-problem induced culled birds were affected by the variable-light intensity lighting program. Expression of tryptophan hydroxylase 2 in the DRN and VTA of variable-light treated birds was lower than that of 5-lx- and 20-lx-treated birds on day 42 (*p* < 0.05). Higher expression of VTA-TH in 5-lx-treated birds than that in 20-lx-, NL-, and variable-light-treated birds suggests the high stress-susceptibility of 5-lx treated birds. Lower VTA-GR expression in 20-lx- and variable-light-treated birds indicates lower stress than that in NL- and 5-lx-treated birds (*p* < 0.05). The VTA-BDNF expression of NL-treated birds was 2.5 fold higher than that of 5-lx-, 20-lx-, and variable-light-treated birds (*p* < 0.05), and variable-light-treated birds showed the lowest level of BDNF expression (*p* < 0.05), suggesting the chronic social defeat stress in NL-treated birds. The result of VTA-Opn4 expression on day 42 suggests the possible role of VTA-Opn4 in broiler welfare through central light perception. Taken together, the variable-light intensity lighting program increased volunteer natural behaviors and physical activity, which may improve footpad condition and leg health of birds, consequently. Performance data including the increased daily weight gain and the lowered feed conversion ratio and results of brain welfare indicator gene expression showed the beneficial effect of the variable-light intensity lighting program on the performance and welfare of commercial broilers.

## 1 Introduction

Light is a critical environmental factor that can affect behaviors, welfare, and production for intensively housed commercial broilers (Alvino et al., 2009; Deep et al., 2010; Olanrewaju et al., 2016; Wu et al., 2022). One of the most noticeable effects of light on birds is the effect of light intensity on birds’ health and behavior (Deep et al., 2010; Blatchford et al., 2012; Olanrewaju et al., 2018). Light intensity has been shown to affect the activity of birds, but most studies have focused on constant light intensities to determine their effect on welfare and performance. Intriguingly, research by Blatchford et al. (2012) showed a strong effect of light intensity contrast on the behavior and health of broilers and suggested that high contrast in light intensity was associated with strong daily rhythms of behavior. In the previous light preference studies, broiler chickens showed preference for different light intensities (Berk, 1995; Prayitno et al., 1997; Raccoursier et al., 2019; Kang et al., 2020). Broiler chickens showed preference for the higher intensity light when they are performing active behaviors but prefer dimmer areas when resting (Newberry et al., 1985; Berk, 1995; Raccoursier et al., 2019; Kang et al., 2020).

Leg health is one of the most prevalent causes of culling and late mortality during grow-out of commercial broilers. It has been suggested that increasing locomotor activity in broilers may improve their welfare (Bizeray et al., 2002; Kristensen et al., 2004; Reiter and Bessei, 2009; Kang et al., 2020). Our previous variable light intensity studies indicate that when birds have a dual light choice, they consumed more feed in the lighter intensity area (20 lux (lx)) than in the lower intensity area (2 lx) (Raccoursier et al., 2019; Kang et al., 2020). There was no significant difference in production parameters (body weight and feed conversion ratio). However, the results of central welfare indicator studies suggest better central welfare in VL intensity-treated birds (Kang et al., 2020).

Most physiological evaluations used in the broilers’ welfare assessment tend to determine negative rather than positive welfare indicators under the assumption that a lack of a negative effect is indicative of well-being (Marcet Rius et al., 2018). Serotonin (5-HT), dopamine (DA), and brain-derived neurotropic factor (BDNF) were suggested as positive indicators in the assessment of animal welfare (Boissy et al., 2007; Polter and Kauer, 2014; Rault et al., 2018; Kang et al., 2020). 5-HT is a major neurotransmitter in the central nervous system (CNS) involved in emotional states caused by stress, pain, or the availability of food (Chamas et al., 1999; Mosienko et al., 2012). In mammals, chronic stress stimulated tryptophan hydroxylase 2 (TPH2: rate-limiting enzyme of 5-HT biosynthesis) gene expression levels in the raphe nuclei of the brainstem (Chamas et al., 1999), suggesting the activation of 5-HT metabolism. A recent animal study showed the increasing of brain 5-HT levels during a novel object test (Ursinus et al., 2013), suggesting an important role of 5-HT in the behavioral responses of animals when confronted with a challenging situation. The amygdalar complex and nucleus accumbens are associated with positive emotional states, and the nucleus accumbens is the terminal site of the DAergic mesolimbic axis from the midbrain VTA (Ikemoto, 2007; Holly and Miczek, 2016). DA-releasing neurons of the VTA are located near the base of the midbrain and play central roles in reward-related and goal-directed behaviors (Morales and Margolis, 2017). The VTA-DAergic neurons are involved in integrating complex inputs to convert multiple signals that influence motivated behaviors (Beier et al., 2015; Bouarab et al., 2019). The avian VTA contains cell bodies that label positively for tyrosine hydroxylase (TH; the rate-limiting enzyme in catecholamine biosynthesis) and have been investigated in broilers as a welfare indicator in the brain (Kang et al., 2020). BDNF is a stress- and activity-dependent neurotrophic factor involved in many activities and modulated by the hypothalamic–pituitary–adrenal (HPA) axis (Jerzemowska et al., 2012; Phillips, 2017; Rault et al., 2018). Animal studies have shown that physical exercise is associated with increased expression of BDNF in the brain and may improve memory performance and reduce depressive symptoms by promoting neurogenesis and neuronal differentiation (Hötting and Röder, 2013; Arosio et al., 2021). In addition, BDNF concentrations in the brain and blood are correlated, offering the opportunity to use peripheral BDNF as a minimally invasive measure of effective enrichment reflecting neural changes. Another important regulator of VTA activity is glucocorticoid (GC), which is synthesized in response to a range of stimuli including stress and is regularly measured in the assessment of animal welfare (Ralph and Tilbrook, 2016). Its action relies on the GC receptor (GR) which translocates into the nucleus upon ligand binding and regulates the transcription of a battery of genes. VTA-GR signaling was suggested to be involved in stress and reward system, regulating the feeding behavior (Daftary et al., 2009; Hensleigh and Pritchard, 2013; Ferrario et al., 2016; Mizoguchi et al., 2021).

Therefore, addressing the central 5-HT, and DA, VTA-GR, and VTA-BDNF in broilers may provide data to aid in understanding possible adaptive behavioral or physiological responses of commercial broilers to the environment. In the present study, we hypothesized that when broilers in commercial houses are provided with the VL intensity lighting environment, it will stimulate birds’ innate natural behavior, causing volunteer movement for consumption of feed and water and consequently improving physical activity and helping leg health and welfare of birds. To test this hypothesis, we have focused on the effects of the VL intensity lighting program on the gene expression of the indicators of natural behaviors, footpad condition, performance, and central welfare in the brain compared to other constant light intensities and natural light.

## 2 Materials and methods

### 2.1 Experimental design, animal housing, and sample preparation

One-day-old broilers (Cobb 700, mixed sex, and 19,200 birds/house) were housed in four commercial broiler houses (Tyson Foods Broiler Welfare Research Farm (BWRF)). Four replicate trials were performed, and each house was composed of four quadrant sections (compartment). Each quadrant of the house was placed with 4,800 chicks with all source flocks equally represented in each quadrant. Birds were raised for 56, 51, 49, and 55 days in trails 1, 2, 3, and 4, respectively. Each house was equipped with standard feeders, waterers, and brooders (12.8 m × 122 m, wood shavings). Two of the houses have a 60-cm-wide strip of clear plastic that runs the length of the houses from 120 to 180 cm high on the sidewall and allows for natural light to enter. The natural light window can be sealed to convert that house to internal illumination. In each trial, four different light intensity lighting programs were installed, and the light intensity (LED) was measured at nine different areas of the house. Averages of light intensity in 5-lx, 20-lx, natural-light (NL), and VL houses were 6.16 ± 0.16 lx, 26.16 ± 0.70 lx, 483.76 ± 42.02 lx, and 2.07/40.4 ± 0.04 lx, respectively. A diet was formulated to meet minimum industry standards (Council, 1994). Light was switched on at 6 a.m. on day 1–3 (23L1D_40 lx), and then on day 4–7, the photoperiod schedule was changed to 20L4D_20 lx. The NL house received supplemented light for maintaining the same photoperiod. On day 7, lighting programs were started for 5-lx, 20-lx, NL, and VL houses (16L8D; light switched on at 6 a.m.). The VL house received about 40 lx light intensity over the feed lines and dimmer light intensity at the sidewalls (2–5 lx). Data on dustbathing holes and other natural behaviors were obtained weekly without interruption of the time schedules. In trials 1 and 3, the brains of the birds in each section were sampled on days 14, 28, and 42. Birds in each section (n = 12/treatment; male) were randomly selected, weighed, and transported to the brain sampling room. Daily body weight gain and feed conversion ratio were obtained from the processing plant at the end of the trial for each house. The guidelines for care and experimental use of animals were followed, and all birds were maintained in accordance with the protocol of Tyson Foods BWRF.

### 2.2 Observation of welfare-related behaviors

#### 2.2.1 Dustbathing holes and daily physical activity

In trial 3, the number of dustbathing holes was counted within six identified areas of the section (4 sections/house) to evaluate the effects of the four different light intensity lighting programs (5 lx, 20 lx, NL, and VL) on the dustbathing behavior. In each section, dustbathing holes as the evidence of dustbathing behavior were counted at 9, 16, and 23 days of age. The number of holes per 10 m^2^ was determined. Data were compared among treatments. In trial 4, daily broiler activity was monitored using a 22-g activity tracker, Animo (www.surepetcare.com, activity and behavior monitor), which monitors animals’ activity and behavior including sleep quality, energy burnt, and shaking *via* tri-axial accelerometer technology. A similar animal activity tracker, FitBark (www.fitbark.com), was used for monitoring animal’s movement in the behavior study (Delgado et al., 2022). At 38 days of age, birds were randomly selected and body weight was measured (n = 16/house; 20 lx and VL house). An Amino tracker was installed for each bird using a commercially available chicken harness and uninstalled at 43 days of age. Average daily activity (joules/day) of each bird was obtained from the installed software. Animo energy calculation is based on an industry standard calculation that takes into account the bird’s weight. The energy burnt is tracked against each movement type for birds.

#### 2.2.2 Footpad condition test

In trial 3, in each section of houses, randomly selected broiler feet (n = 10/section and four sections/house) were evaluated with a scoring range of 0–2. Footpad data were analyzed in a pass and fail manner. Lesion scorings on the feet of birds were given as follows: 0—no evidence of footpad dermatitis (pass), 1—minimal evidence of footpad dermatitis (fail), and 2—evidence of footpad dermatitis (fail). Pass birds have normal color and minimal swelling, and no lesion was found in more than half the area of the central pad. Fail birds have discoloration and swelling, and lesions were found in more than half the area of the central pad. Footpad condition was checked at 28 and 42 days of age, and the percentage of failed birds to the inspected birds was obtained and compared among treatment groups.

#### 2.2.3 Novel object test

In trial 3, the novel object test was conducted for each quadrant (section) of the four houses. The test was carried out every 7 days in the morning, starting at 31 days of age. The test was conducted by placing a novel object in the center of the section by an observer. Observation points were set the same every week. The novel object was a cone-shaped container (30 cm H x 24 cm D) and had identical proportions of green, yellow, and red colors. The observer moved away from the object, and the bird activity was evaluated by counting the number of birds that approached toward the novel object in different timepoints (30 s, 1 min, 5 min, 10 min, and 15 min).

### 2.3 Leg-problem-induced culled birds and mortality

In each section of trials 1 and 3, the accumulated number of birds that were culled because of leg problems and total dead birds were obtained from day 7 to 14, day 7 to 28, and day 7 to 49. To evaluate the effects of different light intensity lighting programs on leg health and total mortality, the number of total dead birds and leg-problem-induced culled birds was obtained on day 7. Leg problems of culled birds included any leg issue that prevented the bird to access feed or water and/or marked a gait score of 2 based on the modified Tyson Foods’ gait scoring system. Mortality was recorded daily, and birds were removed when they died, were unable to move to access feed and water, appeared to be sick or injured, and exhibited signs of lameness (one or both legs splayed and severe hock/paw). Accumulated mortalities were obtained from day 7 to 14, day 7 to 28, and day 7 to 42. The same counting process was used for counting the number of birds which were culled due to leg problems (one leg or two legs). Data were compared among treatments.

### 2.4 Dissection of the dorsal raphe nucleus (DRN) and caudal raphe nucleus (CRN) of the brainstem and ventral tegmental area (VTA) of the midbrain

According to the previous studies on avian species and a chick brain atlas (Kuenzel and Masson, 1988; Kang et al., 2009; Kang et al., 2020), two major 5-HTergic regions in the brainstem, DRN and CRN, and VTA regions were dissected in a cryostat microtome. The dimensions of the dissected section are as follows: 2.5–3 mm (W) x 1–1.5 mm (H) x 2.5–3.0 mm (L) for DRN; 2–2.5 mm (W) x 1–1.2 mm (H) x 2.5–3.0 mm (L) for CRN; and 3–3.5 mm (W) x 2–3 mm (H) x 1–1.2 mm (L) for VTA. The thickness (W, H, and L) of the dissected brain tissue block was proportionally increased from young birds to older birds based on the brain size and structure. Inside the cryostat, the brain areas shown as rectangles were dissected from each flattened brain section using a scalpel handle and blade (#11) and were quickly transferred to TRIzol reagent and then stored at -80°C until total RNA extraction.

### 2.5 RNA isolation and two-step real-time quantitative RT-PCR

Total RNA was extracted from dissected frozen tissues using TRIzol^®^ reagent (Invitrogen Life Technologies, Palo Alto, CA, United States) followed by DNase I treatment and purification of total RNA using the RNeasy Mini Kit (Qiagen, Valencia, CA, United States). The RNA quality and quantity were determined using agarose gel electrophoresis and NanoDrop 1000 (Thermo Scientific, Wilmington, DE, United States). A volume of 2 µg of the total RNA from each sectioned tissue was converted into cDNA with oligo (dT)_16_ primer and SuperScript III reverse transcriptase (Invitrogen, Grand Island, NY, United States), as previously described (Kang and Kuenzel, 2014; Nagarajan et al., 2017; Kang et al., 2017, 2020). The specific oligonucleotide primers were designed using the Primer3 program (http://frodo.wi.mit.edu). A total of five primer sets for chicken TPH2, TH, GR, BDNF, and Opn4 were designed, and conventional RT-PCR was performed for optimizing annealing temperature for each primer set (Table 1). The PCR products were analyzed by agarose gel electrophoresis (3%). Melting curve analysis and PCR efficiency for each selected primer set were validated with the default settings on the ABI 7500 system (Applied Biosystems LLC, Foster, CA, United States). The efficiency of PCR was evaluated by performing a dilution series experiment, and the slope of the standard curve was translated into an efficiency value. The efficiency of the PCR within 95%–100% was accepted for this study. A portion of the cDNA was subjected to real-time quantitative RT-PCR (qRT-PCR) using an ABI 7500 system with Power SYBR Green PCR Master Mix (Invitrogen, Grand Island, NY, United States). Chicken glyceraldehyde 3-phosphate dehydrogenase (GAPDH) and β-actin were used as internal controls. Dissociation curves were constructed at the end of amplification for validating the quality of the data. All qRT-PCR experiments were performed in triplicate, and the values of the average cycle threshold (Ct) were determined, and the delta-Ct scores for gene transcripts in each sample were normalized using the delta-Ct scores for GAPDH/β-actin and expressed as the relative fold change in gene expression using the 2^−ΔΔCt^ method. The gene name, NCBI accession numbers, primer sequences, PCR product size, and annealing temperatures used in the present study are shown in Table 1.

**TABLE 1:** Primers used for RT-qPCR.

Gene	GenBank #	Primer sequence (5′–3′)	Size (bp)	Annealing Tm (°C)
TPH2	NM_001001301.1	F: AGG​ACC​TCC​GCA​GTG​ATC​TA	111	58
		R: CAG​CAT​AAG​CAG​CTG​ACA​ACA		
TH	NM_204805	F: CTT​TGA​TCC​TGA​TGC​TGC​TG	103	56
		R: CCT​CAG​CTT​GTT​TTT​GGC​AT		
GR	NM_001037826	F: GCC​ATC​GTG​AAA​AGA​GAA​GG	95	54
		R: TTT​CAA​CCA​CAT​CGT​GCA​T		
BDNF	NM_001031616	F: GAC​ATG​GCA​GCT​TGG​CTT​AC	167	60
		R: GTT​TTC​CTC​ACT​GGG​CTG​GA		
Opn4	AY036061	F: AAG​GTT​TCG​CTG​TCA​TCC​AGC	128	58
		R: CTG​CTG​CTG​TTC​AAA​CCA​AC		
GAPDH	NM 204305	F: CTT​TGG​CAT​TGT​GGA​GGG​TC	128	58–60
		R: ACG​CTG​GGA​TGA​TGT​TCG​G		
β -actin	L08165	F: CAC​AAT​GTA​CCC​TGG​CAT​TG	158	54–56
		R: ACA​TCT​GCT​GGA​AGG​TGG​AC		

### 2.6 Statistical analyses

Statistical analyses were performed using JMP^®^ 14.0 (SAS Institute Inc., NC). Normal distribution was first tested, and differences among the groups were analyzed using one-way analysis of variance (ANOVA) followed by mean comparison using Tukey’s HSD test at a significance level of *p* < 0.05. Multiple comparisons of group means by Tukey’s HSD test were used to evaluate behavior data, including dustbathing holes, daily physical activity, footpad lesion, and novel object test, accumulated mortality, the number of culled birds, and relative changes in TPH2, TH, GR, BDNF, and Opn4 mRNA expression among treatment groups for each gene. Data are presented as the mean ± SEM. A probability level of *p* < 0.05 was considered statistically significant.

## 3 Results

### 3.1 Effects of different light intensity programs in a commercial broiler house on welfare-related behaviors

**FIGURE 1:**
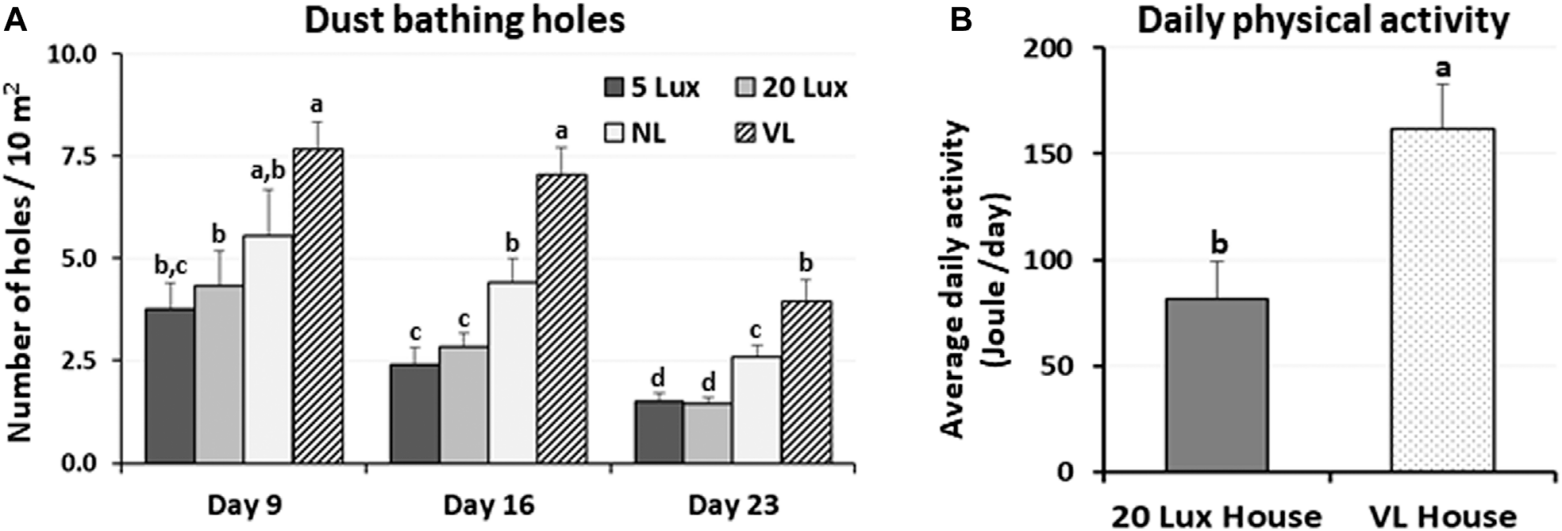
Effects of the four different lighting programs (5 lx, 20 lx, NL, and VL) on the number of dustbathing holes and daily physical activity at different ages. **(A)** In the each section of trial 3, dustbathing holes as the evidence of dustbathing behavior were counted at 9, 16, and 23 days of age. Dustbathing holes were observed in six parts of each section and number of holes per 10 m^2^ was determined. Data (mean ± SEM) were compared among treatments. Different lower-case letters above the bars denote significant differences (*p* < 0.05) among groups, where a>b and a,b is not different from a or b. **(B)** In trial 4, an activity tracker, Animo, was installed on bird using harness at 38 days of age, and uninstalled at 43 days of age (total *n* = 16, 4/sections, 20 lx and VL house). Average daily activities (calorie consumption) for each bird from day 39 to day 42 obtained. Data (mean ± SEM) were compared between 20 lx and VL houses. Different lower-case letters above the bars denote significant differences (*p* < 0.05) between groups.

**FIGURE 2:**
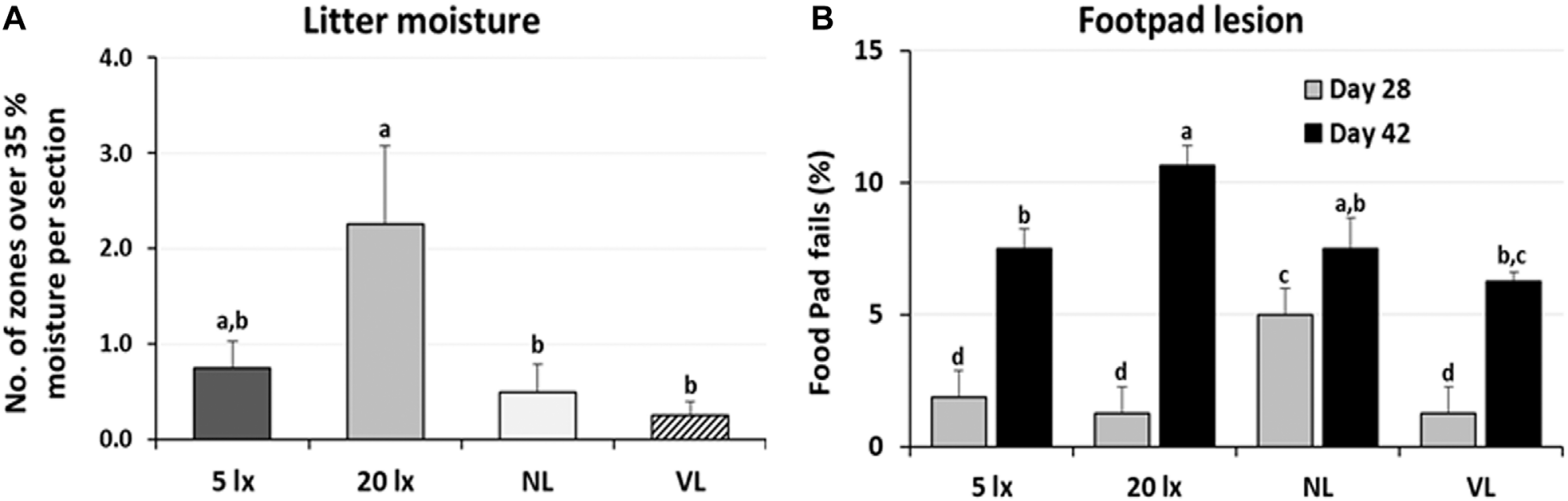
Effects of the four different lighting programs (5 lx, 20 lx, NL, and VL) on the litter moisture and footpad health. **(A)** In trial 1, condition of litter moisture was accessed by the hand-clumping method. When litter begins to retain moisture and moisture content is over 35%, it clumps together. Seven different areas (zones) of each section were tested if the litter clumps by hand. Average of number of zones per section with positive hand-clumping was obtained in each treatment of light programs and compared among treatment groups. **(B)** In trial 3, footpad condition was checked at 28 and 42 days of age in each section of the treatment houses (*n* = 10/section, 4 sections/house). Percentage of footpad failed birds from the inspected birds was obtained and compared among treatment groups. Data (mean ± SEM) were compared among treatments. Different lower-case letters above the bars denote significant differences (*p* < 0.05) among groups, where a>b>c. b,c is not different from b or c.

**FIGURE 3:**
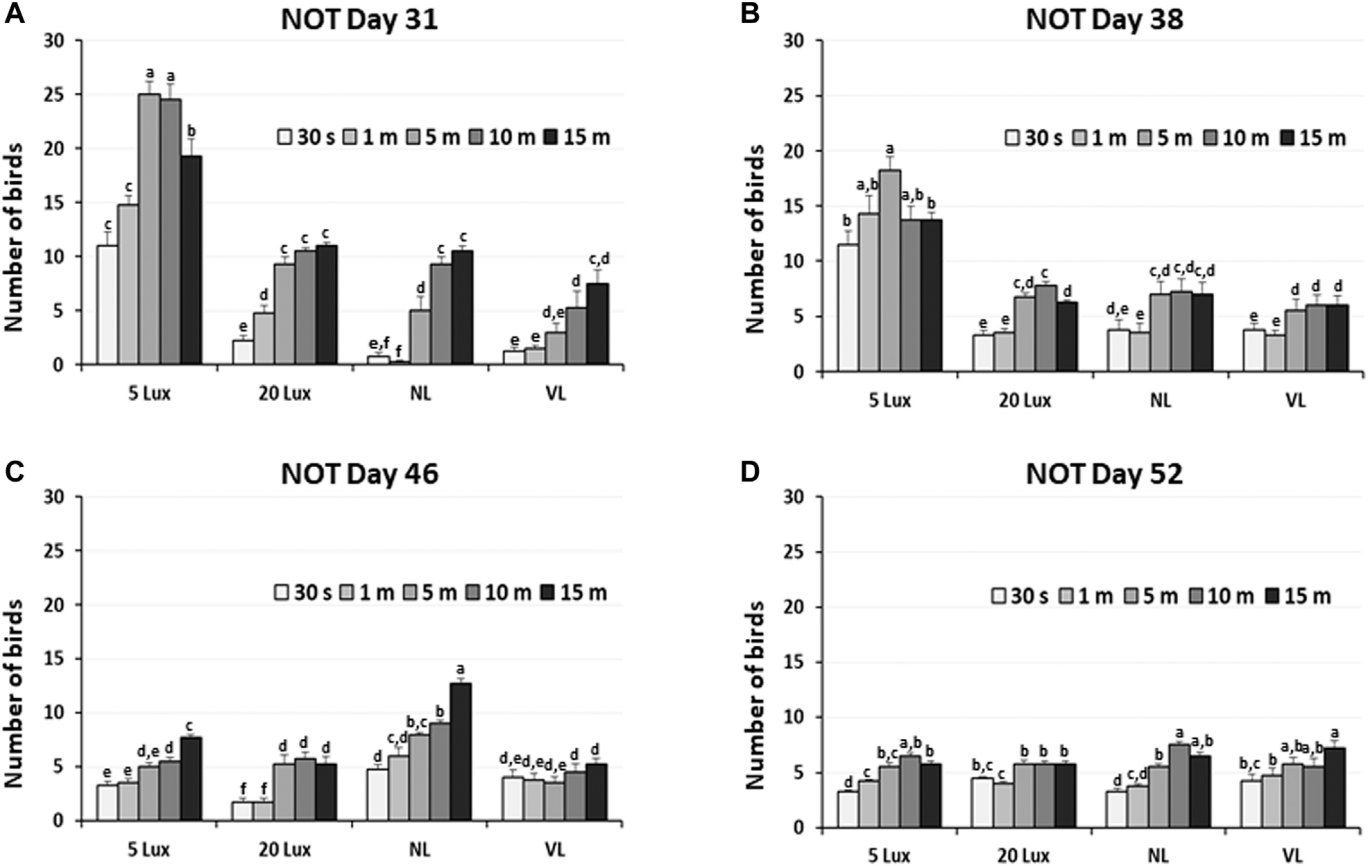
Novel object test in four different lighting program houses (5 lx, 20 lx, NL, and VL) at different ages **(A)** age of day 31, **(B)** age of day 38, **(C)** age of day 46, and **(D)** age of day 52. Numbers of birds approaching the novel object were obtained in the sequential time points (30 sec, 1, 5, 10, and 15 min) in each section of the houses, and data (mean ± SEM) were compared among treatments. Different lower-case letters above the bars denote significant differences (*p* < 0.05) among groups, where a>b>c>d>e>f and a,b is not different from a or b.

The dustbathing behavior made holes on the floor of commercial broiler houses. Weekly counting of the numbers of holes in each section of the house was performed and compared (Figure 1A). The numbers of dustbathing holes in the NL and VL houses were higher than those in the 5-lx house on day 9 (2 days light treatment) (*p* < 0.05). The numbers of dustbathing holes in both NL and VL houses were higher than those in 5-lx and 20-lx houses (*p* < 0.05), and in the VL house, the number of dustbathing holes was higher than that in the NL house (*p* < 0.05). To evaluate the effect of light intensity programs in 20-lx and VL houses on the daily activity of broilers, Animo, an activity tracker, was used in the 20-lx and VL houses of trial 4 (n = 16). Average body weights (mean ± SEM) of birds randomly selected for activity tracking in 20-lx and VL houses were 2.622 ± 0.061 and 2.752 ± 0.055, respectively. Average daily consumed energy by moving activity was obtained for 4 days (from day 39 to day 42) (Figure 1B). For their moving activity, VL-treated birds used twice as much energy per day as 20-lx-treated birds (*p* < 0.05). The percentage of footpad-failed birds to the inspected birds (see Section 2.2.2 for grading scale) was obtained and compared among treatment groups. On day 28, the NL house had a significantly higher footpad-failed percentage than the other groups (Figure 2). On day 42, the 20-lx house had the higher number of footpad-failed birds than 5-lx and VL houses (*p* < 0.05). The novel object test was performed on four different light intensity lighting program houses (5 lx, 20 lx, NL, and VL) at different ages. The numbers of birds approaching toward the novel object were obtained in the sequential timepoints (30 s, 1 min, 5 min, 10 min, and 15 min) in each section of the houses. On day 31, the first testing day of the novel object, there was a significant difference in the number of birds approaching toward the novel object in the 5-lx house compared to other houses (*p* < 0.05). The numbers of approached birds in the 5-lx house in different timepoints were higher than those in other houses on days 31 and 38 (Figures 3A, B). On days 46 and 52, the difference in the number of approached birds among houses was decreased (Figures 3C, D).

### 3.2 Effects of different light intensity programs on broiler performance in a commercial broiler house

**FIGURE 4:**
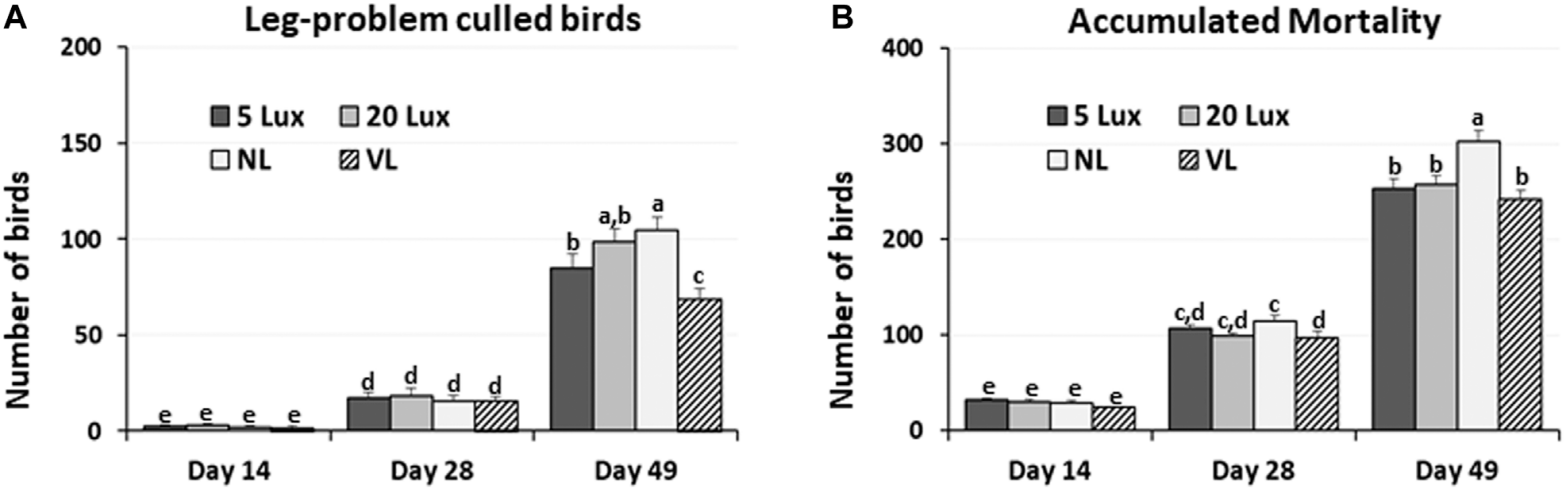
Accumulated number of birds that were culled by leg-problems and mortality in four different lighting programs (5 lx, 20 lx, NL, and VL) at different ages. **(A)** In each section of trial 1 and 3, the accumulated number of birds that were culled because of leg-problems and total dead birds were obtained from day 7 to day 14 (Day 14), day 7–28 (Day 28) and day 7–49 (Day 49). **(B)** Accumulated mortality from trial 1 and was obtained from day 7 to day 14 (Day 14), day 7–28 (Day 28) and day 7–49 (Day 49). Data (mean ± SEM) were compared among treatments. Different lower-case letters above the bars denote significant differences (*p* < 0.05) among groups, where a>b>c>d>e>f and a,b is not different from a or b.

In each section of trials 1 and 3, the accumulated number of birds that were culled because of leg problems and total dead birds in each section were obtained from day 7 to 14, day 7 to 28, and day 7 to 49. Leg problems of culled birds included any leg issue that prevented the bird to access feed or water and/or had a gait score of 2. The number of culled birds on day 49 decreased by 19%, 30%, and 34% in VL-treated birds compared to 5-lx-, 20-lx-, and NL-treated birds (Figure 4A). Total mortality was 25% lower in VL-treated birds than NL-treated birds (Figure 4B). There were slight decreases in mortality in the VL house compared to 5-lx and 20-lx houses on days 28 and 49 (*p* > 0.05). The average daily weight gain of the birds in the VL house was 4.3, 1.2, and 4.1% higher than that in 5-lx, 20-lx, and NL houses, respectively (Table 2). From the feed conversion ratio (FCR) of four trials (total house numbers of 20-lx and VL house were 5), the average FCR of 20-lx-treated and VL-treated birds was 1.953 and 1.908, respectively. The average FCR of VL-treated birds was 2.2% lower than that of 20-lx- and NL-treated birds.

**TABLE 2:** Effects of different light programs on the production performance of commercial broilers.

Trial/treatment	Daily weight gain (g)	Feed conversion ratio (FCR)
Trial 1 (56 days)		
5-lux house	67.13	1.958
20-lux house	66.27	1.977
NL house	65.68	1.996
VL house	67.31	1.932
Trial 2 (51 days)		
5-lux house	66.36	1.858
20-lux house	64.23	1.842
NL house	63.96	1.871
VL house	66.54	1.821
Trial 3 (49 days)		
5-lux house	63.87	1.922
20-lux house	66.18	1.905
NL house	61.64	1.993
VL house	66.27	1.890
Trial 4 (55 days)		
20-lux house	68.95	1.970
20-lux house	67.63	2.070
NL house	68.67	1.944
VL house	70.62	1.953

### 3.3 Differential effects of different light intensity programs on 5-HTergic activity in dorsal raphe nucleus (DRN) and caudal raphe nucleus (CRN) of the brainstem of commercial male broiler chickens

**FIGURE 5:**
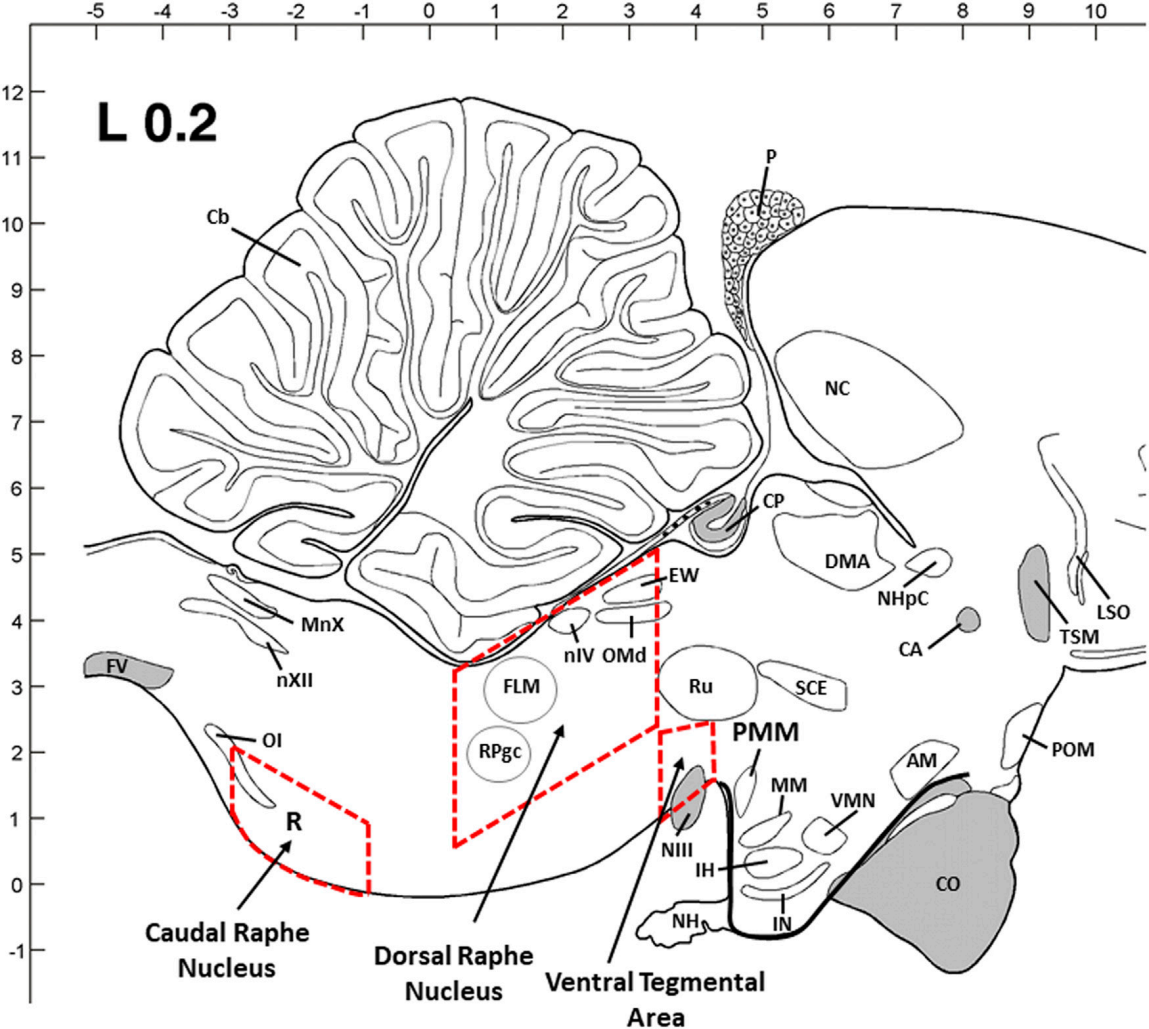
Sagittal view of three dissection areas of the chicken brain [dorsal raphe nucleus (DRN) and caudal raphe nucleus (CRN) of the brainstem and ventral tegmental rea (VTA) of the midbrain]. Dimensions of the dissected tissues are coronal with 2.5–3 mm (W) x 1–1.5 mm (H) x 2.5–3.0 mm (L) for DRN, 2–2.5 mm (W) x 1–1.2 mm (H) x 2.5–3.0 mm (L) for CRN, and 3–3.5 mm (W) x 2–3 mm (H) x 1–1.2 mm (L) for VTA. The thickness (W, H, and L) was adjusted proportionally from young birds to older birds based on the brain size and structure. Abbreviations: AM: anterior medial hypothalamic nucleus; CA: anterior commissure; Cb: cerebellum; CO: optic chiasma; CP: posterior commissure; DMA: dorsomedial nucleus; EW: Edinger–Westphal nucleus; FLM: medial longitudinal fasciculus; FV: ventral fasciculus; IH: inferior hypothalamic nucleus; IN: infundibular hypothalamic nucleus; LSO: lateral septal organ; MM: medial mammillary nucleus; MnX: nucleus motorius dorsalis nervi vagi; NC: caudal neostriatum; NH: neurohypophysis; NHpC: nucleus of the hippocampal commissure; NIII: oculomotor nerve; nIV: trochlear nerve nucleus; nXII: hypoglossal nerve nucleus; OI: inferior olivary nucleus; OMd: dorsal oculomotor nucleus; P: pineal gland; POM: medial preoptic nucleus; PVN: paraventricular nucleus; RPgc: nucleus of caudal pontine reticular gigantocellular; Ru: red nucleus; SCE: stratum cellular externum; TSM: septopallio-mesencephalic tract; VMN: ventromedial hypothalamic nucleus.

**FIGURE 6:**
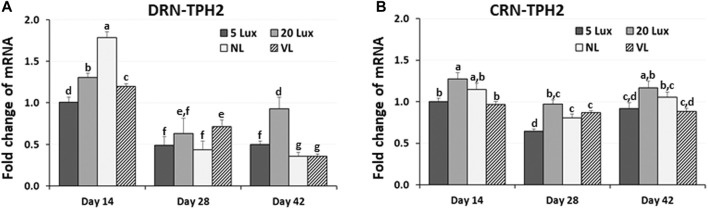
Expression changes of TPH2 in **(A)** the dorsal raphe nucleus (DRN) and **(B)** caudal raphe nucleus (CRN) of brainstem. Light intensity programs were changed at day 7–5, 20, 480 lx (NL house), 2–5 lx/40 lx (VL house). Brains of male bird were sampled on days 14, 28 and 42 (*n* = 12/section, 4 sections/house). DRN and CRN of the brainstem from each bird were dissected as described in Figure 5. Total RNA was extracted and used for real time RT-qPCR for TPH2 expression. Data were set as the relative fold changes of expression levels using the ΔΔCt method with GAPDH and β-actin as internal controls. Data (mean ± SEM) were expressed from a value set for 1.0 for 5 lx birds at 14 days of age. Different lower-case letters above the bars denote significant differences (*p* < 0.05) among groups, where a>b>c>d>e>f>g and a,b is not different from a or b.

As an indicator of serotonergic activity, the TPH2 mRNA level was determined in two brainstem areas, DRN and CRN, at 14, 28, and 42 days of age (Figure 5). Changes in TPH2 gene expression were determined in the DRN and CRN of the brainstem and were compared among treatments at 14, 28 and 42 days of age (Figure 6). In the DRN of the broiler brainstem, TPH2 gene expression was significantly higher in NL-treated birds than in other light treatment groups at 14 days of age (*p* < 0.05). TPH2 expression in 5-lx-treated birds was significantly lower than that in the 20-lx- and VL-treated birds on day 14 (Figure 6A). However, on day 28, DRN-TPH2 expression of all treatment birds decreased and was insignificant. On day 42, TPH2 expression in the DRN was the lowest in NL- and VL-treated birds (*p* < 0.05) and highest in 20-lx-treated birds compared to other treatment groups. In the CRN of the brainstem, TPH2 expression was significantly lower in 5-lx- and VL-treated birds on day 14 than that in the 20-lx-treated birds (*p* < 0.05) (Figure 6B). There was significant lower expression of TPH2 in the CRN of 5-lx-treated birds than in 20-lx-treated birds on days 28 and 42. On days 28 and 42, TPH2 expression of each treatment group of birds became less significant (*p* < 0.05).

### 3.4 Regulation of welfare marker and melanopsin genes in the ventral tegmental area (VTA) by different light intensity programs in a commercial broiler house

**FIGURE 7:**
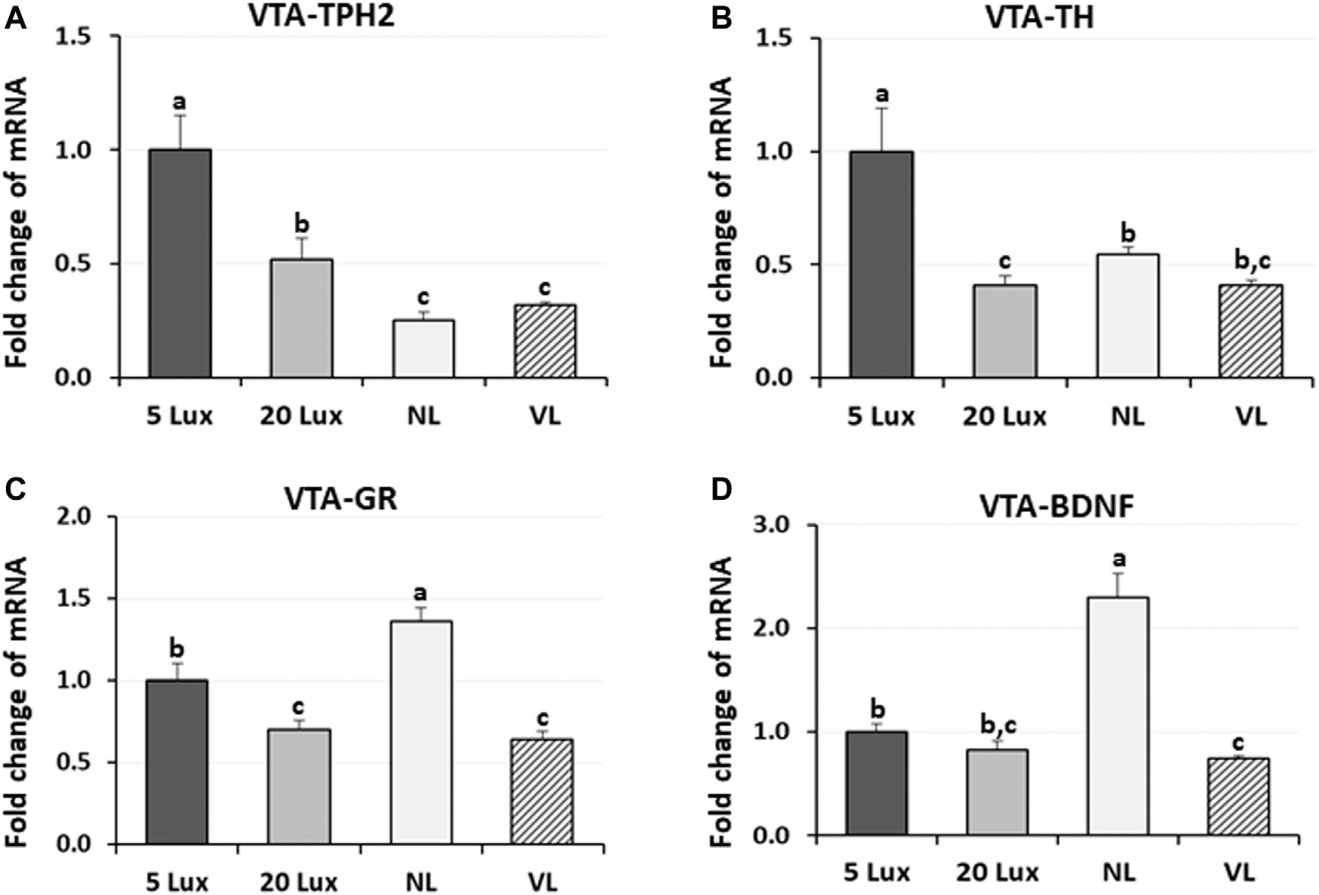
Regulation of VTA welfare related marker genes. Expression changes of **(A)** TPH2, **(B)** tyrosine hydroxylase (TH, the rate-limiting enzyme of dopamine biosynthesis), **(C)** glucocorticoid receptor (GR), and **(D)** brain-derived neurotropic factor (BDNF) mRNA in the ventral tegmental area (VTA) of birds at 42 days of age were measured in four different lighting program houses. Light intensity programs were changed at day 7–5, 20, 480 lx (NL house), 2–5 lx/40 lx (VL house). Brains of male bird were sampled on day 42 (*n* = 12/section, 4 sections/house). VTA of the brainstem from each bird was dissected as described in Figure 5. Total RNA was extracted and used for RT-qPCR. Data were set as the relative fold changes of expression levels using the ΔΔCt method with GAPDH and β-actin as internal controls. Data (mean ± SEM) were expressed from a value set for 1.0 for 5 lx birds for each gene. Different lower-case letters above the bars denote significant differences (*p* < 0.05) among groups, where a>b>c and b,c is not different from b or c.

To evaluate the long-term effects of different light intensity programs on the previously identified welfare marker genes, expression of TPH2, TH, GR, and BDNF genes was determined at 42 days of age (Figure 7). The expression of TPH2 mRNA in the VTA of NL-treated and VL-treated birds was lower than that of 5-lx-treated and 20-lx-treated birds (*p* < 0.05). The TPH2 mRNA level in the VTA of 5-lx-treated birds was the highest compared to other light-treated birds (*p* < 0.05). TH mRNA expression, an indicator of DAergic activity, was the highest in 5-lx-treated birds compared to other light-treated birds (*p* < 0.05), and there were no significant differences in TH expression between 20-lx- and VL-treated birds and between NL- and VL-treated birds at 42 days of age. VTA-GR is the stress modulator and involved in the stress-induced plasticity and functioning of VTA (Douma and de Kloet, 2020). Lower VTA-GR expression was observed in 20-lx- and VL-treated birds than that in 5-lx- and NL-treated birds (*p* < 0.05), indicating that the stress is higher in NL-treated birds than in 20-lx- and VL-treated birds (*p* < 0.05). The over-activation of GR in NL-treated birds may stimulate a dysfunctional reward system in VTA and plays an important role in the pathogenesis (Mizoguchi et al., 2021). BDNF is a social stress-related neurotrophic factor in the VTA (Berton et al., 2006). The VTA-BDNF expression of NL-treated birds was 2.5 fold higher than that of 5-lx-, 20-lx-, and VL-treated birds (*p* < 0.05), and VL-treated birds showed the lowest level of BDNF expression (*p* < 0.05), suggesting the chronic social defeat stress in NL-treated birds compared to 5-lx-, 20-lx-, and VL-treated birds (Fanous et al., 2010). These results suggest that social stress can cause long-term neuroadaptations involving both GR and BDNF in the VTA.

**FIGURE 8:**
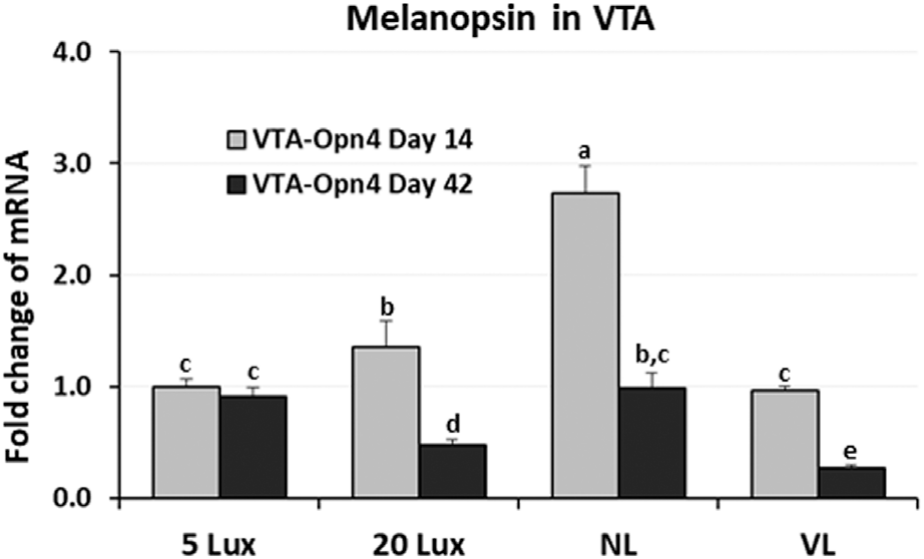
Expression changes of melanopsin (Opn4) mRNA in the VTA of birds at 14 and 42 days of age. Light intensity programs were changed at day 7–5, 20, 480 lx (NL house), 2–5 lx/40 lx (VL house). Brains of male bird were sampled on days 14 and 42 (*n* = 12/section, 4 sections/house). VTA of the midbrain from each bird was dissected as described in Figure 5. Total RNA was extracted and used for RT-qPCR. Data were set as the relative fold changes of expression levels using the ΔΔCt method with GAPDH and β-actin as internal controls. Data (mean ± SEM) were expressed from a value set for 1.0 for 5 lx birds at 14 days of age. Different lower-case letters above the bars denote significant differences (*p* < 0.05) among groups, where a>b>c and b,c is not different from b or c.

For the first time, the avian VTA was suggested as an important area of the midbrain of birds involved in the light perception by melanopsin (Opn4, photoreceptor) and might be involved in the welfare of poultry (Kang, 2021). To evaluate the long-term effects of light intensity programs on the previously identified melanopsin (photoreceptor), the expression of Opn4 gene was determined at 14 and 42 days of age (Figure 8). On day 14, Opn4 expression was the highest in NL-treated birds (*p* < 0.05) and Opn4 expression in 5-lx- and VL-treated birds was significantly lower than that in 20-lx- and NL-treated birds (*p* < 0.05). On day 42, after 5 weeks of light treatment, the expression in VL-treated birds became the lowest among the groups and there was no significant difference between 5-lx- and NL-treated birds, and the expression of Opn4 mRNA in 20-lx-treated birds was intermediate between that in 5-lx/NL- and VL-treated birds (*p* < 0.05).

## 4 Discussion

### 4.1 Effects of different light intensity programs on the broiler behaviors in a commercial broiler house

Environments recognized by birds are either threatening their survival or causing homeostatic disruption, resulting in the behavioral responses and physiological impact on birds. Different lighting programs in commercial broiler houses may influence diverse behavioral and physiological impact on birds. The effects of four different light intensity lighting programs on broiler welfare-related behaviors were evaluated in the present study. Dustbathing is performed by many avian species and is considered a comfort behavior (Vestergaard et al., 1997; Louton et al., 2016). Dustbathing holes as the evidence of dustbathing behavior were counted in three timepoints, which indicates that the VL intensity lighting program in the commercial broiler house has a stimulating effect on the dustbathing behavior and dustbathing is the most observed natural activity in VL-treated birds. As the birds got older, evidence of dustbathing decreased, which could be due to age or reduced friable litter.

An activity tracker, Animo, was able to monitor the daily physical activity of birds at an older age. Results showed significant stimulation of birds’ daily physical activity in VL-treated birds compared to 20-lx-treated birds. The number of dustbathing holes in the NL house was intermediate between the 5-lx/20-lx and VL houses, suggesting that the bright light in NL also stimulates locomotive activity of NL-treated birds. In fact, several studies have indicated that the stimulatory effect of bright light on broilers increased locomotor activity (Newberry et al., 1988; Blatchford et al., 2009). Footpad condition is critical and associated with birds’ leg health and moving activity. The footpad-failed percentage in each section was the highest in the 20-lx house and was the lowest in the VL house on day 42 (*p* < 0.05), indicating that there is a possibility of stimulated natural movement by the VL intensity lighting program, as observed in the dustbathing holes and activity tracking studies, which may be beneficial to footpad health.

Environment lighting has long been recognized as a factor that can change the perceived atmosphere of the environment, and fear is an adaptive emotional response to potentially harmful stimuli and serves to protect animals from injury (Steimer, 2002; 2009; Vasdal et al., 2018).

VTA DAergic neurons are involved in the extinction mechanism of fear responses, and the increase in DA release has been observed in the nucleus accumbens during fear extinction in the fear extinction learning study (Badrinarayan et al., 2012; Salinas-Hernandez et al., 2018).

In the novel object test (Figure 3), we observed a rapid increase in the fear-sensing mechanism in 5-lx-treated birds as these birds have less access to the novel object than other light-treated birds, suggesting that the experienced fear of the novel object was different in the four different light intensity lighting programs we tested. When fear response is not normal or different, birds may have an impaired ability to sense mental alertness. It may be speculative that the fear-sensing mechanism of 5-lx-treated birds was impaired on day 31, at least for an individual’s ability to function, but it became adapted in the later test on days 46 and 52. This will be discussed in detail in Section 4.3 with the result of VTA-TH expression in 5-lx-treated birds.

### 4.2 Effects of different light intensity programs on the leg health and performance of commercial broilers

To increase performance and productivity, commercial broilers are often raised in houses that are dimly illuminated on a near-continuous basis. Several studies on broilers addressing broiler leg health suggest that the stimulatory effect of bright light on locomotor activity can improve their leg condition and thus their welfare (Newberry et al., 1988; Shields et al., 2005; Blatchford et al., 2009). In the present study, fascinating and engrossing results were observed in leg health and performance of the VL house.

Within-treatment house comparison in each trial showed the consistency in the lowest number of leg-problem-induced culled birds and total mortality after light treatments, suggesting that the increased natural movement behaviors, as observed in this study, appear to improve the leg health of VL-treated birds. The improved leg health may contribute to the reduction in mortality in the VL house. In addition, we observed the highest daily weight gain (DWG) and the lowest FCR in the VL house in each trial (Table 2) consistently. These results indicate the economic and welfare benefit of the VL intensity lighting program in broiler production.

### 4.3 Differential effects of light intensity programs on 5-HTergic activity in dorsal raphe nucleus (DRN) and caudal raphe nucleus (CRN) of the brainstem of commercial male broiler chickens

It was suggested that the acceptable welfare of animals is not simply the absence of negative experiences, but rather it is primarily the presence of positive experiences (Boissy et al., 2007; Marcet Rius et al., 2018). Animals use the sense of vision to examine the surrounding area and locate food sources. Recent studies showed that the 5-HTergic system in the raphe nuclei of the brainstem was involved in this behavioral choice decision which was affected by light (Filosa et al., 2016; Kang et al., 2020). The stress-induced activation of TPH2 expression was observed in the raphe nuclei of mammals, and 5-HT metabolism and turnover were increased in the brain (Inoue et al., 1994; Chamas et al., 1999). The elevation of TPH2 mRNA expression in the raphe nuclei was reported to be involved in the emotional conditions, such as depression in mammals and avian species (Bach-Mizrachi et al., 2008; Kang et al., 2020). To address the welfare of birds under different lighting programs in commercial broiler houses, we measured TPH2 expression in DRN and CRN of the brainstem as an indicator of 5-HTergic activity in the brain. Results showed that there was a significantly higher expression of TPH2 in the DRN of 20-lx- and NL-treated birds than VL-treated birds on day 14 and in the DRN of 20-lx-treated birds than VL-treated birds on day 42, indicating that VL-treated birds experience lower stress than 5-lx- and 20-lx-treated birds on day 42. Results of TPH2 expression in CRN suggest that 5-HTergic activities in the DRN are more associated with the light intensity-related physiological response of birds.

### 4.4 Regulation of welfare-indicating genes and melanopsin (Opn4) in the ventral tegmental area (VTA) by different light intensity programs in a commercial broiler house

In the VL house, birds rested and slept in the dim light area of the house and actively fed and drank water in the 40-lx light intensity area, which may provide the appropriate environment for the homeostatic control of their autonomic nervous system (ANS). The ANS allows animals to adapt to their environment by equilibrating sympathetic (SNS) and parasympathetic nervous systems (PNS) (Briefer et al., 2015; Daniela et al., 2022). 5-HT and DA are critical neurotransmitters to regulate PNS and SNS, respectively. Compensatory changes in 5-HTergic and DAergic activities were suggested to restore the balance of the brain’s homeostatic mechanisms, and these compensatory changes take days to develop (Andrews et al., 2015; Pratelli et al., 2017). Recent studies found that the VTA of the midbrain contains cell bodies of mesolimbic DAergic neurons as well as the 5-HTergic system in mammals and avian species (Kang et al., 2009; Carkaci-Salli et al., 2011; Chaudhury et al., 2013; Grace, 2016). The upregulated 5-HTergic and DAergic activities in VTA of 5-lx-treated birds suggest that the higher synthesis of these two positive welfare indicators in 5-lx-treated birds indicates the high stress-susceptibility and may be regulated by the compensatory mechanism as suggested in the previous reports (Belujon and Grace, 2015; Grace, 2016; Welford et al., 2016; Kang et al., 2020).

There is a growing body of evidence that stress affects learned fear response (Krishnan, 2014; Maren and Holmes, 2016; Steimer, 2002). GCs were suggested for detecting animal welfare (Ralph and Tilbrook, 2016), and our previous study indicated that there was no consistency in corticosterone (CORT) levels in different timepoints, and no long-term effect of lighting programs on CORT was observed in circulating blood (Kang et al., 2020). One of the major actions of GCs is to regulate the transcription of its primary target gene, GR, through genomic GC response elements (GREs) by directly binding to DNA or tethering onto other DNA-binding transcription factors. These GR primary targets trigger physiological and pathological responses of GCs. Therefore, we used the GR gene expression in VTA (VTA-GR) to investigate the effect of different variable light intensity lighting programs on the welfare of birds as described in other animal studies (Daftary et al., 2009; Mizoguchi et al., 2021; Tran et al., 2022). The lower level of GR in 20-lx- and VL-treated birds indicates that the VTA-DAergic system is necessary to initiate fear extinction and reveal a crucial role of DA neurons in this form of safety learning.

Physical exercise is an evidence-based treatment strategy to improve broiler’s leg health as discussed previously (Bizeray et al., 2002; Kristensen et al., 2004; Reiter and Bessei, 2009). Several mechanisms may explain the positive impact of physical exercise, including an increase in neurotrophic support. One proposed theory is that the mechanism of action of exercise could involve the neurotrophic pathway, especially the BDNF (Liu and Nusslock, 2018). Animal studies have shown that physical exercise is associated with the increased expression of BDNF in the hippocampus, which may improve memory performance and reduce depressive symptoms by promoting neurogenesis and neuronal differentiation (Hötting and Röder, 2013; Arosio et al., 2021). However, unlike BDNF in the hippocampus, the activation of VTA-BDNF was suggested to be involved in the long-term social defeat stress, and BDNF gene deletion in the VTA attenuated stress-induced behaviors, such as social avoidance in mice (Berton et al., 2006; Krishnan et al., 2007; Fanous et al., 2010). Taken together, the results of welfare-related gene expression in the brain of commercial broilers against stress indicate the beneficial effects of the VL intensity lighting program on broilers’ welfare.

Many studies provide evidence that light can affect the central physiology of animals independent of the retinal function (Underwood et al., 1984; Wade et al., 1988; Fernandes et al., 2013). VTA was suggested as an important area of the midbrain involved in the light perception by melanopsin (Opn4, photoreceptor) and might be involved in the avian welfare (Kang, 2021). The result of long-term effects of light intensity programs on Opn4 expression at 42 days of age in the present study suggests that Opn4 in VTA may be involved in the direct perception of light intensity information for physiological adaptation of birds.

## 5 Conclusion

The findings of the present study show an extensive understanding of effects of variable light (VL) intensity lighting programs on the welfare and performance of broiler chickens. Here, we evaluated the effects of constant light intensities (5 and 20 lx), natural light (480 lx), and VL intensity lighting programs (2–5 lx/40 lx) on the behavior, performance, and welfare indicators in commercial broiler chickens. We observed an increased broiler dustbathing behavior and better performance in the VL intensity lighting house, suggesting a beneficial effect of the VL intensity lighting program on broiler natural exercise. This lighting program stimulates voluntary walking behavior for consuming feed/water and taking rest as well as improves leg health and performance, providing the valuable information on how to improve broilers’ welfare and performance in a commercial broiler farm using the VL intensity lighting program.

## Data Availability

The original contributions presented in the study are included in the article/Supplementary Materials; further inquiries can be directed to the corresponding author.
